# Management of Nitrogen Through the Use of Leaf Color Chart (LCC) and Soil Plant Analysis Development (SPAD) or Chlorophyll Meter in Rice Under Irrigated Ecosystem

**DOI:** 10.1100/tsw.2004.137

**Published:** 2004-09-13

**Authors:** Debtanu Maiti, D. K. Das, Tanmoy Karak, Mahua Banerjee

**Affiliations:** Department of Agricultural Chemistry and Soil Science, Faculty of Agriculture, Bidhan Chandra Krishi Viswavidyalaya, Mohanpur, Nadia-741252, West Bengal, India; ^2^ Department of Soil and Water Sciences, College of Resources and Environmental Sciences, China Agricultural University, Beijing-100094, P.R., China; ^3^ Division of Agronomy, Indian Agricultural Research Institute, Pusa, New Delhi-110012, India

**Keywords:** N-use efficiency, LCC, SPAD, chlorophyll meter, rice

## Abstract

A field experiment was conducted in a farmers field in the district of Nadia, West Bengal, India to study the management of N through leaf color chart (LCC) and soil plant analysis development (SPAD) or chlorophyll meter in rice (cv. IET-4094) during the Kharif (wet season) of 2001—2002 and 2002—2003 by taking the treatment combinations based on different levels of N at fixed schedule and through LCC and SPAD. The experimental soil (0—15 cm) had pH 7.33; organic C 0.43%; available N 408.70 kg ha^−1^; available P 6.92 kg ha^−1^; and available K 66.31 kg ha^−1^. The results of LCC and SPAD or chlorophyll meter for the N management in rice show that values of both LCC and SPAD significantly increased with an increasing level of N. The mean values of LCC and SPAD varied from 3.19—5.31 and 27.36—39.26, respectively, in rice. The results show that the amount of N can be saved as 20—42.5 and 27.5—47.5 kg N ha^−1^ through the use of LCC and SPAD in rice over the fixed-timing N treatment T_7_ where 150 kg N ha^−1^ was applied in three (3) splits without reduction in the yield. The SPAD- and LCC-treated N plot showed higher N-use efficiency over fixed-scheduling N treatment in rice. The results further show that SPAD value of 37 and LCC value of 5 have been proved to be superior treatments over SPAD (35) and LCC (4) for the best management of N in rice in an Inceptisol.

## INTRODUCTION

For achieving high yields of rice (*Oryza sativa* L.), farmers in many parts of the world tend to apply N in excess of the requirements. This is particularly true in rice-growing areas of the Indo-Gangetic plain in

Eastern India and it leads to further lowering of N fertilizer recovery efficiency (RE), which is already not more than 50%[1]. When N application is nonsynchronized with crop demand, N losses from the soil-plant system are large, resulting in low N fertilizer use efficiency. Hence, plant need-based application of N is crucial for achieving high yield and N-use efficiency. The chlorophyll meter, also known as SPAD (soil plant analysis development), can quickly and reliably assess the N status of a crop based on leaf area. It has been successfully used for rice[2]. The high cost of the chlorophyll meter keeps it out of reach of many Asian farmers. The leaf color chart (LCC) is an inexpensive alternative to the chlorophyll meter. Like the chlorophyll meter or SPAD, the critical color shade on the LCC needs to be determined to guide N applications. Fertilizer N-use efficiency of irrigated rice is relatively low due to rapid loses of applied N through volatilization and denitrification in the soil. The leaf N content is closely related to photosynthetic rate[3] and the N concentration on a dry weight basis of the topmost fully expanded leaf has been used as an index to determine the N topdressing.

Use of this approach in developing countries of Asia is very limited. The chlorophyll meter or SPAD and LCC provide a simple, quick, and nondestructive method for estimating N of rice leaves. Very limited research works are available so far to establish LCC and SPAD for rice in Eastern India, particularly in West Bengal. Therefore, the present investigation was conducted to evaluate need-based N management strategies for rice using LCC and SPAD with the following objectives: (1) to save N without decreasing yield of rice, (2) to avoid expenditure on soil test for the recommendation of N fertilizers, and (3) to find out the relative efficiency of LCC and SPAD for the N economy as well as increasing yield.

## EXPERIMENTAL METHODS

A field experiment was conducted in a farmer's field of Ranaghat Block II in the district of Nadia (22º57′ N latitude and 88º20′ E longitude, average altitude of 7.8 m above mean sea level), West Bengal for rice (in *Kharif*), replicated thrice in a randomized block design. The IET-4094 for rice was taken as a test crop. The recommended levels of N (60 kg $ha^{-1}$) in one plot, and $P_2O_5$ and $K_2O$ in all plots, were applied for growing rice. Each field was divided into 12 subplots carrying the following treatments. The levels of N were $T_1$: 0 kg $ha^{-1}$; $T_2$: 60 kg $ha^{-1}$; $T_3$: 80 kg $ha^{-1}$; $T_4$: 100 kg $ha^{-1}$; $T_5$: SPAD (35) with no basal N; $T_6$: SPAD (35) with basal N @ 10 kg $ha^{-1}$; $T_7$: SPAD (37) with no basal N; $T_8$: SPAD (37) with basal N @ 10 kg $ha^{-1}$; $T_9$: LCC (4) with no basal N; $T_{10}$: LCC (4) with basal N @ 10 kg $ha^{-1}$; $T_{11}$: LCC (5) with no basal N; and $T_{12}$: LCC (5) with no basal N @ 10 kg $ha^{-1}$. Recommended doses of phosphate (30 kg/ha) and potash (30 kg/ha) were applied to all plots. The N was applied on the basis of SPAD and LCC values @ 15 kg $ha^{-1}$ at 21–42 days after transplanting (DAT); 15 kg $ha^{-1}$ at 43–63 DAT, and 10 kg $ha^{-1}$ at 64–84 DAT.

The chlorophyll meter and LCC were used for measurements on five topmost fully expanded leaves. The LCC (supplied by International Rice Research Institute, Philippines) and SPAD (Model, CCM-200) readings were taken at 7-day intervals at a specified time. An average of six readings of leaves was taken in each plot.

All the data of rice for 2 years were pooled statistically and then the relevant data were statistically analyzed for Duncan's Multiple Range Test (DMRT); correlation and multiple regression etc. following the standard procedures [4] and [5].

### Formulae Used

$$AE = \Delta \text{ kg grain/kg N applied} \quad (1)$$

$$RE = (\Delta \text{ kg uptake/fertilizer N applied}) \times 100 \quad (2)$$

$$PE = \Delta \text{ kg grain}/\Delta \text{ kg N uptake} \quad (3)$$

where AE = agronomic efficiency, RE = recovery efficiency, PE = physiological efficiency, Δ = difference between treatment plot and control plot.

$$AE = RE \times PE \text{ (units of AE, RE, and PE, kg kg}^{-1}\text{ N)} \quad (4)$$

$$Pfp = Y/N_r = (Y_0 + \Delta Y)/N_r = (Y_0/N_r) + (\Delta Y/N_r) = (Y_0/N_r) + AE \quad (5)$$

Pfp = partial factor productivity, $Y_0$ = yield without N, $N_r$ = level of N.

## RESULTS AND DISCUSSION

### LCC and SPAD Values vs. Grain Yield

The LCC values increased significantly with an increase of N levels (Table 1). The recorded LCC values did not show any particular trend with the progress of crop growth, but mostly the LCC values increased up to 63 DAT of rice and thereafter, decreased at 84 DAT. The range of LCC values were 2.97–5.70 irrespective of treatments and progress of crop growth. The SPAD or chlorophyll meter values increased significantly with increasing levels of N (Table 2). The trend of changes in SPAD was almost similar to that of LCC, however, the SPAD values increased significantly up to 42 DAT, then decreased at 84 DAT, though it did not maintain any particular trend. The range of SPAD values were 27.31–41.76 irrespective of treatments and progress of crop growth. The SPAD-based N application was comparable and superior to other treatments. The present findings also supported the results obtained by Mahender Kumar et al.[6]. Singh et al.[7] indicated that with the application of 30 kg N $ha^{-1}$ each time, the SPAD value fell below the critical value of 37.5, which resulted in application of 90 kg N $ha^{-1}$, which produced rice yields equivalent to those with 120 kg N $ha^{-1}$ applied in three splits. They also suggested that using a SPAD value of 35 was inadequate for two rice cultivars because it resulted in application of only 60 kg N $ha^{-1}$ and thus low yields, which also supports the results of the present investigation.

The mean LCC and SPAD values were positively and significantly correlated at all growth stages with mean grain yield of rice (Table 3). The correlation coefficient (r) values between SPAD and LCC at different growth stages were found significant and positively correlated at all growth stages of rice. The "r" values of grain yield with LCC ranged from 0.479*–0.744** while that of SPAD ranged from 0.827**–0.941**. The significant positive correlations of these parameters indicate that the topdressing of N can be practiced based on the LCC and SPAD. The SPAD showed higher correlation over LCC to achieve the greater yield, indicating an increased N-use efficiency significantly through the use of chlorophyll meter over LCC. But the LCC can also be used for N topdressing as it is low cost and easy to handle in the field as compared to SPAD, which is most expensive and requires technical skill for its operation. The LCC is now well accepted by farmers in developing countries. The mean LCC and SPAD values varied from 3.19–5.31 and 27.36–39.26, respectively.

### Grain Yield and N Uptake

An increase in grain yield from applied N ranged from 3.92–4.95 t $ha^{-1}$ (Table 4) depending on treatment as compared to control, no N application (2.03 t $ha^{-1}$). Grain yields in fixed-schedule N treatments averaged 4.17 t $ha^{-1}$. The largest yield of 4.95 t $ha^{-1}$ was obtained in $T_8$ with SPAD (37)-based along with basal N @ 10 kg $ha^{-1}$ plot receiving total applied N of 72.50 kg $ha^{-1}$. The SPAD (35)–based N plot produced a grain yield that is statistically at par with LCC (4) and LCC (5)–based N plot. The LCC-based N plot produced a grain yield that was not significantly different from that obtained with fixed-timing N treatment $T_4$ where N was applied at 100 kg $ha^{-1}$, but LCC-based N treatment saved up to 2,042.5 kg N $ha^{-1}$ as compared to $T_4$, without reduction in yield. The SPAD-based N produced significantly higher or at

**Table 1**
**Effect of Different Treatments of N on LCC Readings in Rice (Pooled Mean of 2001–2002 and 2002–2003)**

| Treatments | 21 DAT | 42 DAT | 63 DAT | 84 DAT |
|---|---|---|---|---|
| $T_1$ | $3.23^{j,\dagger}$ | $3.18^{f}$ | $3.37^{f}$ | $2.97^{f}$ |
| $T_2$ | $4.61^{d}$ | $4.50^{d,e}$ | $4.35^{e}$ | $3.84^{e}$ |
| $T_3$ | $4.89^{c}$ | $5.38^{a,b,c}$ | $5.13^{c}$ | $4.91^{a,b}$ |
| $T_4$ | $5.34^{a}$ | $5.60^{a,b}$ | $5.32^{b}$ | $4.96^{a}$ |
| $T_5$ | $3.83^{h}$ | $5.31^{a,b,c}$ | $4.64^{d}$ | $4.30^{d}$ |
| $T_6$ | $4.31^{e}$ | $3.37^{f}$ | $4.51^{d}$ | $4.32^{d}$ |
| $T_7$ | $4.00^{f}$ | $5.54^{c,d,e}$ | $5.11^{c}$ | $4.69^{b,c}$ |
| $T_8$ | $5.13^{b}$ | $5.70^{a}$ | $5.37^{a,b}$ | $4.54^{c,d}$ |
| $T_9$ | $3.74^{i}$ | $4.98^{b,c,d}$ | $4.55^{d}$ | $4.29^{d}$ |
| $T_{10}$ | $3.96^{f}$ | $3.31^{f}$ | $4.25^{e}$ | $3.99^{e}$ |
| $T_{11}$ | $3.89^{g}$ | $4.28^{e}$ | $5.47^{a}$ | $4.97^{a}$ |
| $T_{12}$ | $4.91^{c}$ | $5.29^{a,b,c}$ | $5.48^{a}$ | $5.02^{a}$ |
| SEm (±) | 0.0087 | 0.1025 | 0.0229 | 0.0415 |

† Within a column, means followed by the same letter are not significantly different at the 0.05 level of probability by DMRT.

Levels of N: $T_1$ = 0 kg $ha^{-1}$, $T_2$ = 60 kg $ha^{-1}$, $T_3$ = 80 kg $ha^{-1}$, $T_4$ = 100 kg $ha^{-1}$, $T_5$ = SPAD (35) with no basal N, $T_6$ = SPAD (35) with basal N @ 10 kg $ha^{-1}$, $T_7$ = SPAD (37) with no basal N, $T_8$ = SPAD (37) with basal N @ 10 kg $ha^{-1}$, $T_9$ = LCC (4) with no basal N, $T_{10}$ = LCC (4) with basal N @ 10 kg $ha^{-1}$, $T_{11}$ = LCC (5) with no basal N, and $T_{12}$ = LCC (5) with basal N @ 10 kg $ha^{-1}$.

**Table 2**
**Effect of Different Treatments of N on SPAD Readings in Rice (Pooled Mean of 2001–2002 and 2002–2003)**

| Treatments | 21 DAT | 42 DAT | 63 DAT | 84 DAT |
|---|---|---|---|---|
| $T_1$ | $27.41^{f,\dagger}$ | $27.93^{i}$ | $26.77^{d}$ | $27.31^{g}$ |
| $T_2$ | $33.86^{d}$ | $35.95^{f}$ | $34.26^{c}$ | $32.50^{f}$ |
| $T_3$ | $37.21^{a}$ | $36.70^{e}$ | $36.21^{b}$ | $34.46^{d,e}$ |
| $T_4$ | $37.35^{a}$ | $37.68^{d}$ | $36.93^{b}$ | $35.82^{b,c}$ |
| $T_5$ | $33.19^{e}$ | $37.67^{d}$ | $36.23^{b}$ | $35.54^{c}$ |
| $T_6$ | $35.79^{b}$ | $33.91^{h}$ | $36.62^{b}$ | $35.34^{c,d}$ |
| $T_7$ | $34.83^{c}$ | $40.68^{b}$ | $39.08^{a}$ | $37.44^{a}$ |
| $T_8$ | $37.38^{a}$ | $41.76^{a}$ | $40.24^{a}$ | $37.64^{a}$ |
| $T_9$ | $34.75^{c}$ | $36.73^{e}$ | $36.34^{b}$ | $35.23^{c,d}$ |
| $T_{10}$ | $34.86^{c}$ | $34.39^{g}$ | $34.56^{c}$ | $34.12^{e}$ |
| $T_{11}$ | $34.79^{c}$ | $35.95^{f}$ | $36.48^{b}$ | $35.23^{c,d}$ |
| $T_{12}$ | $37.05^{a}$ | $38.22^{c}$ | $37.48^{b}$ | $36.63^{b}$ |
| SEm (±) | 0.1057 | 0.0596 | 0.2192 | 0.1384 |

Means and levels the same as in Table 1.

**Table 3**

**Simple Correlation Matrix Showing the Relationship of LCC and SPAD Values at Different Growth Period of Rice with Grain Yield**

| Parameter | $LCC_{21}$ | $LCC_{42}$ | $LCC_{63}$ | $LCC_{84}$ | $SPAD_{21}$ | $SPAD_{42}$ | $SPAD_{63}$ | $SPAD_{84}$ | Grain Yield |
|---|---|---|---|---|---|---|---|---|---|
| $LCC_{21}$ | 1.000 | | | | | | | | |
| $LCC_{42}$ | 0.607* | 1.000 | | | | | | | |
| $LCC_{63}$ | 0.669** | 0.682** | 1.000 | | | | | | |
| $LCC_{84}$ | 0.592* | 0.607* | 0.921** | 1.000 | | | | | |
| $SPAD_{21}$ | 0.804** | 0.578* | 0.821** | 0.829** | 1.000 | | | | |
| $SPAD_{42}$ | 0.583** | 0.771** | 0.805** | 0.708** | 0.751** | 1.000 | | | |
| $SPAD_{63}$ | 0.574* | 0.600** | 0.832** | 0.784** | 0.840** | 0.914** | 1.000 | | |
| $SPAD_{84}$ | 0.511* | 0.595** | 0.819** | 0.818* | 0.832** | 0.894** | 0.970** | 1.000 | |
| Grain yield | 0.497* | 0.479* | 0.774** | 0.764** | 0.856** | 0.827** | 0.918** | 0.941** | 1.000 |

* Significant at the 0.05 probability level and ** significant at the 0.01 probability level.

$LCC_{21}$, $LCC_{42}$, $LCC_{63}$, and $LCC_{84}$ are LCC values at 21, 42, 63, and 84 DAT, respectively.

$SPAD_{21}$, $SPAD_{42}$, $SPAD_{63}$, and $SPAD_{84}$ are SPAD values at 21, 42, 63, and 84 DAT, respectively.

par yield and saved 27.5–47.5 kg N $ha^{-1}$ as compared to fixed-timing N treatment $T_4$ where 100 kg N was applied in three splits.

From the results presented in Table 4, it was proposed to take the threshold value for SPAD- and LCC-based N (37 and 5, respectively), instead of taking 35 and 4 in rice growing under Eastern Indian conditions which supported the results obtained by Singh et al.[7]. This threshold appears to be sufficient to achieve potential yield levels of rice cultivars. It has also been suggested that different threshold SPAD values may be used for different varieties[8].

The total N uptake (Table 4) was proportional to total N rates and the highest N uptake was observed in the treatment $T_8$ where N was applied on the basis of SPAD (37) along with basal N @ 10 kg $ha^{-1}$ followed by LCC (5)–based and fixed-timing N plot where N was applied at 100 kg $ha^{-1}$ in three splits. The SPAD-based N application proved better over LCC or fixed-scheduling split which might be due to better utilization of N, suggesting that savings in fertilizer N can be accomplished without sacrificing yield using the chlorophyll meter–based N management strategy. Similar views were also reported by Maiti and Das[9] and Peng et al.[10]. LCC and SPAD were also successfully used by Hussain et al.[11]. Mahender Kumar et al.[6] also reported that the grain yield of rice increment was 45 and 43% over the control plot with SPAD-N and N @ 135 kg $ha^{-1}$, respectively. Maiti and Das[9] also suggested that the amount of N can be saved upto 25 kg and 20 kg $ha^{-1}$ without decreasing the yield of rice by the use of SPAD and LCC, respectively, as compared to the treatment where 100 kg N $ha^{-1}$ was applied.

## N-Use Efficiency: AE, RE, PE, and Pfp

The results (Table 4) suggested that the AE and PE were greatest (46.03 and 81.86, respectively) in the treatment $T_5$ with SPAD (35)–based N application followed by SPAD (37)–based N application treatment. The highest RE (68.60) was observed in the treatment $T_9$ with LCC (4)–based N along with no basal N which was statistically at par with SPAD (37)–based N application along with basal N at 10 kg $ha^{-1}$. The AE and PE in the $T_5$, SPAD (35)–based N plot was 46.64–89.19 and 5.75–81.86%, greater than treatments with fixed N splits, respectively. The RE in the treatment $T_9$ LCC (4)–based N plot was

**Table 4**

**Effect of Different Treatments of N on N Application (kg $ha^{-1}$), Grain Yield (t $ha^{-1}$), Total N Uptake (kg $ha^{-1}$), AE, RE in %, PE, and Pfp in Rice (Pooled Mean of 2001–2002 and 2002–2003)**

| Treatments | N Application (kg $ha^{-1}$) | | | Total N Applied | Grain Yield | Total N Uptake | AE | RE | PE | Pfp |
|---|---|---|---|---|---|---|---|---|---|---|
| | Basal | MT | PI | | | | | | | |
| $T_1$ | 0 | 0 | 0 | 0 | $2.03^{g,\dagger}$ | $19.68^{h}$ | — | — | — | — |
| $T_2$ | 30 | 15 | 15 | 60 | $3.92^{f}$ | $44.03^{g}$ | $31.39^{f}$ | $40.80^{c}$ | $77.41^{b}$ | 65.22 |
| $T_3$ | 40 | 20 | 20 | 80 | $4.13^{e}$ | $56.15^{d,e}$ | $26.25^{g}$ | $45.71^{c}$ | $57.47^{e,f}$ | 51.63 |
| $T_4$ | 50 | 25 | 25 | 100 | $4.47^{d}$ | $62.64^{b,c}$ | $24.33^{g}$ | $42.97^{c}$ | $56.63^{e,f}$ | 44.63 |
| $T_5$ | SPAD (35), no basal N | | | 52.50 | $4.45^{d}$ | $49.22^{f}$ | $46.03^{a}$ | $56.23^{b}$ | $81.86^{a}$ | 84.70 |
| $T_6$ | SPAD (35), basal N @ 10 kg $ha^{-1}$ | | | 57.50 | $4.55^{c,d}$ | $54.93^{e}$ | $43.77^{a,b}$ | $61.25^{b}$ | $71.45^{c}$ | 79.07 |
| $T_7$ | SPAD (37), no basal N | | | 62.50 | $4.68^{b,c}$ | $54.11^{e}$ | $42.42^{b,c}$ | $55.14^{b}$ | $77.33^{a,b}$ | 74.90 |
| $T_8$ | SPAD (37), basal N @ 10 kg $ha^{-1}$ | | | 72.50 | $4.95^{a}$ | $72.53^{a}$ | $39.10^{d}$ | $68.40^{a}$ | $56.16^{f}$ | 67.10 |
| $T_9$ | LCC (4), no basal N | | | 57.50 | $4.45^{d}$ | $59.13^{c,d}$ | $42.02^{b,c}$ | $68.60^{a}$ | $61.19^{e}$ | 77.32 |
| $T_{10}$ | LCC (4), basal N @ 10 kg $ha^{-1}$ | | | 62.50 | $4.55^{c,d}$ | $57.70^{d,e}$ | $40.26^{c,d}$ | $60.83^{b}$ | $66.16^{d}$ | 72.74 |
| $T_{11}$ | LCC (5), no basal N | | | 72.50 | $4.60^{b,c,d}$ | $62.31^{b,c}$ | $35.40^{e}$ | $58.76^{b}$ | $60.26^{e,f}$ | 63.40 |
| $T_{12}$ | LCC (5), basal N @ 10 kg $ha^{-1}$ | | | 80.00 | $4.75^{b}$ | $65.30^{b}$ | $33.96^{e}$ | $57.04^{b}$ | $59.38^{e,f}$ | 59.34 |
| SEm (±) | | | | | 0.0263 | 0.6040 | 0.4056 | 0.9651 | 0.7424 | — |

$^{\dagger}$ Within a column, means followed by the same letter are not significantly different at the 0.05 level of probability by DMRT.

AE = Δ kg grain/kg N applied; RE = (Δ kg uptake/Fertilizer N applied) × 100; PE = Δ kg grain/Δ kg N uptake.

Δ = Difference between treatment plot and control plot; AE = RE × PE (units of AE, RE, and PE, kg $kg^{-1}$ N); Pfp = $Y/N_r = (Y_0 + \Delta Y)/N_r = (Y_0/N_r) + (\Delta Y/N_r) = (Y_0/N_r) + AE$.

Pfp = partial factor productivity; $Y_0$ = yield without N; $N_r$ = level of N.

59.18–67.65% greater than treatments with fixed N splits. The same trend of AE, RE, and PE was also observed by Peng et al.[10]. The Pfp from applied nutrients is a useful measure of nutrient-use efficiency because it provides an index that quantifies total economic yield relative to utilization of all nutrient resources. The Pfp of N was highest (84.70) in the treatment $T_5$ where N was applied based on SPAD (35) with no basal N (Table 4) due to greater yield with smaller N rate. Pfp proved to be a useful index for the diagnosis of constraints to improve N-use efficiency in the farmer's fields because it reflects both AE and the balance between the indigenous N supply and applied N. Similar results were also observed by Cassman et al.[12].

The simple correlation matrix (Table 5) indicated that the AE, RE, and PE have been found to be significantly correlated with grain yield and N uptake by rice. The grain yield of rice was significantly and positively correlated with N uptake (0.927**), AE (0.861**), RE (0.934**), and PE (0.786**).

The grain yield of rice has been found to be significantly varied with the N uptake, AE, RE, and PE (Table 6). The N uptake, AE, RE, and PE togetherly contributed 99.2% of the variability towards the grain yield of rice.

**Table 5**
**Simple Correlation Matrix Showing the Relationship of N Uptake by Rice, AE, RE, and PE with Grain Yield**

| Parameter | N Uptake | AE | RE | PE | Grain Yield |
|---|---|---|---|---|---|
| N uptake | 1.000 | | | | |
| AE | 0.651* | 1.000 | | | |
| RE | 0.858** | 0.922** | 1.000 | | |
| PE | 0.535* | 0.876** | 0.731* | 1.000 | |
| Grain yield | 0.927** | 0.861** | 0.934** | 0.786** | 1.000 |

* Significant at the 0.05 probability level and ** significant at the 0.01 probability level.

**Table 6**

**Multiple Regression Equation of N Uptake by Rice, AE, RE, and PE with Grain Yield**

| Parameter | Regression Equation | Coefficient of Determination ($R^2$) |
|---|---|---|
| Grain yield vs. N uptake, AE, RE, and PE | $GY = 1.0699 + 0.0490\ N_u + 0.0395\ AE - 0.0201\ RE + 0.0039\ PE$ | 0.992* |

GY = Grain yield, $N_u$ = N uptake.

* Significant at the 0.01 probability level.

## Benefit:Cost Ratio

The mean grain yield of rice was highest (4.95 t $ha^{-1}$) in the treatment $T_8$ where N was applied based on SPAD (37) with basal N @ 10 kg $ha^{-1}$ (Table 7). The highest benefit:cost ratio (1.82) was observed in the treatment $T_7$ where N was applied based on SPAD (37) with no basal N followed by the treatment $T_8$ (1.80). The results suggested that farmers will benefit by N management in rice through the use of SPAD over LCC and fixed-scheduling N splits. The results also suggested that the threshold value for SPAD in Eastern Indian conditions should be 37 to achieve more benefit from rice.

## CONCLUSIONS

Grain yield of and N-use efficiency can be increased in rice through the use of LCC and SPAD. N topdressing can be practiced in the field of rice on the basis of LCC and SPAD values by taking the threshold limit of 5 and 37, respectively, in Eastern India under irrigated ecosystem. Through the use of LCC and SPAD, 20–42.5 and 27.5–47.5 kg N $ha^{-1}$ can be saved, respectively, over that of the highest level of fixed-timing N applications. Use of LCC and SPAD is economically viable and cost effective.

## ACKNOWLEDGMENTS

The authors are grateful to the Indian Council of Agricultural Research (ICAR), New Delhi for sponsoring this National Agricultural Technology Project (ICAR/NATP/R-W/7B-3, PSR-25), which was successfully completed in collaboration with International Rice Research Institute (IRRI), Philippines and Rice-Wheat Consortium (RWC), New Delhi.

**TABLE 7**
**Effect of Different Treatments on Benefit:Cost Ratio for Rice (Pooled Mean of 2001–2002 and 2002–2003)**

| Treatment | Grain Yield Range (t $ha^{-1}$) | Mean Yield (t $ha^{-1}$) | Added Yield Over Control (t $ha^{-1}$) | Value of Added Yield (Rs. $ha^{-1}$) | Cost of Added Inputs (Rs. $ha^{-1}$) | Added Profit Over Control (Rs. $ha^{-1}$) | Benefit:Cost Ratio |
|---|---|---|---|---|---|---|---|
| $T_1$ | 1.9–2.2 | 2.04 | — | — | — | — | — |
| $T_2$ | 3.7–4.1 | 3.92 | 1.88 | 9,964.00 | 4,326.00 | 5,638.00 | 1.31 |
| $T_3$ | 3.9–4.4 | 4.14 | 2.10 | 11,130.00 | 4,633.00 | 6,497.00 | 1.41 |
| $T_4$ | 4.2–4.8 | 4.47 | 2.37 | 12,534.50 | 5,192.00 | 7,342.50 | 1.42 |
| $T_5$ | 4.2–4.7 | 4.45 | 2.42 | 12,799.50 | 4,833.00 | 7,966.50 | 1.65 |
| $T_6$ | 4.3–4.8 | 4.55 | 2.52 | 13,329.50 | 4,885.00 | 8,444.50 | 1.73 |
| $T_7$ | 4.4–4.9 | 4.69 | 2.65 | 14,045.00 | 4,987.00 | 9,058.00 | 1.82 |
| $T_8$ | 4.7–5.0 | 4.95 | 2.92 | 15,449.50 | 5,541.00 | 9,908.50 | 1.80 |
| $T_9$ | 4.2–4.7 | 4.45 | 2.42 | 12,799.50 | 4,785.00 | 8,014.50 | 1.68 |
| $T_{10}$ | 4.3–4.8 | 4.57 | 2.53 | 13,409.00 | 4,937.00 | 8,472.00 | 1.72 |
| $T_{11}$ | 4.4–4.8 | 4.60 | 2.57 | 13,594.50 | 5,191.00 | 8,403.50 | 1.62 |
| $T_{12}$ | 4.6–4.9 | 4.75 | 2.72 | 14,389.50 | 5,381.00 | 9,008.50 | 1.68 |

Levels of N: same as for Table 1.

Minimum support price (MSP) of rice = Rs. 5300.00 $t^{-1}$, urea = Rs. 4.80 $kg^{-1}$, labor charge = Rs. 60.00 $labor^{-1}$, irrigation = Rs. 300.00 $ha^{-1}$ $irrigation^{-1}$, and cost of plant protection chemicals (PPC) = Rs. 1000.00 $ha^{-1}$ $season^{-1}$.

## BIOSKETCHES

**Debtanu Maiti** is a Ph.D. student whose field of specialization is soil chemistry, soil fertility, and plant nutrition. Sri Maiti has published about nine research papers relating to the above fields during the tenure of his Ph.D. research program.

**D.K. Das**, Professor in the Department of Agricultural Chemistry and Soil Science, has 24 years experience in teaching on the undergraduate and postgraduate level, and research relating to soil chemistry, soil fertility, and plant nutrition. Prof. Das has published about 100 original research papers in national and international journals of repute and has also published six books from Kalyani Publishers, India. http://myprofile.cos.com/dkdas

**Tanmoy Karak** has published about ten papers in the field of soil chemistry and plant nutrition. Dr. Karak has been nominated as a visiting Soil Scientist for Indo-China academic exchange program during the year 2003–2005.

**Mahua Banerjee** is a Ph.D. student in Agronomy and is pursuing her Ph.D. research work at the Indian Agricultural Research Institute, New Delhi. During her research period, she has published about five research papers in national and international journals of repute.
